# Linc00152 promotes proliferation in gastric cancer through the EGFR-dependent pathway

**DOI:** 10.1186/s13046-015-0250-6

**Published:** 2015-11-04

**Authors:** Jianping Zhou, Xiaofei Zhi, Linjun Wang, Weizhi Wang, Zheng Li, Jie Tang, Jiwei Wang, Qun Zhang, Zekuan Xu

**Affiliations:** Department of General Surgery, The First Affiliated Hospital of Nanjing Medical University, Nanjing, Jiangsu P.R. China; Department of Gastrointestinal Surgery, The Affiliated Yixing Hospital of Jiangsu University, Yixing, Jiangsu P.R. China; Department of General Surgery, The Affiliated Hospital of Nantong University, Nantong, Jiangsu P.R. China

**Keywords:** LncRNA, Xenograft transplantation, RNA pull-down, EGFR, PI3K

## Abstract

**Background:**

Linc00152 has been identified highly associated with the tumorigenesis and development of gastric cancer, however, the detailed mechanism of Linc00152 involved still remains unclear.

**Methods:**

RT-PCR and western blot were used to detect the expression of Linc00152 and EGFR. The CCK8 and EDU assay was employed to measure cell proliferation while xenotransplantation technology was applied in BALB/C nude mice. The interaction between lncRNA and target protein was investigated by RNA pull-down and RNA immunoprecipitation assay.

**Results:**

In this study, we first confirmed the upregulation of cytoplasmic expressed Linc00152 in 72 pair tissues of gastric patients. A suppression of cell proliferation and tumor growth was obtained in MGC803 and HGC-27 cells treated with Linc00152 shRNA. RNA pull-down and RIP assay revealed that Linc00152 could directly bind with EGFR which caused an activation of PI3K/AKT signaling.

**Conclusion:**

We first found that Linc00152 could promote tumor growth through EGFR-mediated PI3K/AKT pathway which may serve as potential targets for therapy in the future.

## Background

Gastric cancer (GC) is one of the most common malignant tumors and the second leading cause of cancer-related mortality worldwide [1, 2]. Despite recent improvements in surgery and chemotherapy, gastric cancer remains a very high morbidity and mortality [3, 4]. Therefore, the further investigation of potential mechanism, prognostic biomarkers or therapeutic targets of gastric cancer is essential for the development of useful indicators that aid novel effective therapies for gastric cancer [1, 5, 6].

With the development of whole-genome sequencing technology, it was determined that less than 2 % of the mammalian genome is in protein-encoded regions and the remainder is in noncoding RNAs (ncRNAs) [7]. Among them are long noncoding RNAs (lncRNAs) with the transcripts of greater than 200 nucleotides with no or little protein coding function [8]. Recent studies have identified multiple functional effects of lncRNAs involving in multiple progression of human cancers including regulating gene expression through modulation of chromatin remodeling, controlling of gene transcription, post-transcriptional mRNA processing, protein function or localization and intercellular signaling [9–11]. Furthermore, Researchers also identified that several lncRNAs could be modified epigenetically including methylation, ubiquitination, miRNA-induced regulation though a network [12, 13].

In this study, we mainly focused on the reported lncRNA entitled Linc00152. It has been reported that Linc00152 was participated in cell cycle arrest, apoptosis, epithelial to mesenchymal transition (EMT), cell migration and invasion in gastric cancer [14]. Further exploring found that Linc00152 could also act as a circulating biomarker for the diagnosis of gastric cancer [15]. All the identified results revealed that Linc00152 played a crucial role in the pathogenesis of gastric cancers; however, the detailed mechanism of Linc00152 involved as well as the target protein or signaling still remain unclear.

In the present study, we found that Linc00152 was up-regulated in GC tissues than that in corresponding non-tumor tissues. We also confirmed that Linc00152 could regulate cell growth both in vitro and in vivo. In addition, we demonstrated that cytoplasmic Linc00152 could directly bind with EGFR which caused a constitutive expression and activation of EGFR and EGFR signaling pathway.

## Methods

### Clinical samples and cell cultures

The clinical data was obtained from 72 cases of patients who underwent gastric cancer radical resection surgery during January 2010 to December 2014 at Yixing People Hospital Affiliated to Jiangsu University (Wuxi, China). No patient had the history of exposure to either radiotherapy or chemotherapy before the surgery, and no other co-occurrence cancers was diagnosed. This study was approved by the Ethical Committee of Jiangsu University, and every patient had written informed consent.

The human gastric cancer cell lines used in this study were obtained from the American Type Culture Collection (ATCC) (Manassas, VA, USA). All the cell lines were maintained in an atmosphere of 5 % CO_2_ and grown in suitable medium (Thermo, Beijing, China) supplemented with 10 % fetal bovine serum (Thermo, Beijing, China). Cell lines authentication was performed by STR profiling before initiation of this study.

### Quantitative real time polymerase chain reaction (qRT-PCR)

Quantitative real time polymerase chain reaction (qRT-PCR) was performed to determine the expression levels of Linc00152 and other related mRNAs. Total RNAs were extracted from frozen tissues and cell lines using TRIzol reagent as described by the manufacturer’s protocol (Invitrogen Life Technologies Co, CA, USA). GAPDH was used as an internal control. RT-PCR was performed using ABI Prism 7900HT (Applied Biosystems, CA, USA) according to the direction of the reagents. 2^-ΔΔCT^ was used to calculate the expression results obtained from ABI 7900HT. The mRNA expression of Linc00152 in human tissues was normalized to 18S.

### Western blot

The proteins were extracted as previously described [16]. Equal amounts of cellular proteins were separated by 10 % SDS-PAGE and visualized using an ECL kit (Millipore, MA, USA).

### Cell proliferation assay

The ability of cells proliferation was assayed using CCK-8 (Dojin Laboratories, Kumamoto, Japan) and EDU (Millipore, Massachusetts, America) according to the manufacturer’s instructions. The mock and infected cells were seeded at a density of 1 × 10^4^ cells/well in 96-well flat-bottom and respectively cultured for CCK-8 and EDU assays according to the protocol provided by manufacture. CCK8 was detected at 0, 24, 48 and 72 h. The EDU assay was performed after the cells were cultured for 48 h.

### In situ hybridization

Cells were fixed and permeablized using xylenes, ethanol and protease to allow for biotin-labeled probe access. Slides were boiled in pretreatment buffer for 15 min and rinsed in water. Next, the probe was hybridized to the lncRNA at 38 °C for 2 h. After this, the preamplifier was hybridized to the target probes at 30 °C and amplified with 6 cycles of hybridization followed by 2 washes. Cells were counter-stained to visualize signal. Finally, slides were DAB stained, dehydrated with 100 % ethanol and xylene, and mounted in a xylene-based mounting media.

### The subcutaneous xenotransplantation model

Animal care and euthanasia were approved by the Jiangsu University animal studies committee. Cells (1 × 10^6^) stably Linc00152 shRNA were subcutaneously implanted into the bilateral axillas of 10 BALB/C nude mice in each group. Tumors were measured every week after implantation, and the volume of each tumor was calculated (length × width^2^ × 0.5). All mice were sacrificed 5 weeks afterwards, and the xenografts were peeled off subcutaneously.

### RNA pull-down and RNA Immunoprecipitation (RIP)

The biotin-labeled lncRNA was transcribed with a Biotin RNA Labeling Mix (Roche, CA, USA) and the T7 RNA polymerase (Roche, CA, USA), treated with RNase-free DNase I (Roche, CA, USA) and purified with an RNeasy Mini Kit (Qiagen, Hilden, Germany). Protein extracted from MGC803 was mixed with biotinylated RNA. 60 μL washed streptavidin agarose beads (Invitrogen Life Technologies, CA, USA) was then added to each binding reaction and washed. The associated proteins were resolved by SDS-PAGE, and specific bands were excised and analyzed by mass spectrometry. Proteins in bands were eluted and digested. Digests were analyzed by Orbitrap Velos Pro LC/MS system (Thermo Scientific, CA, USA). Data was analyzed by Proteome Discoverer and the resulting peak lists were used for searching the NCBI protein database with the Mascot search engine.

RIP assay was performed by using EZ-Magna RIP™ RNA-Binding Protein Immunoprecipitation Kit (Millipore, MA, USA) according to the manufacturer’s instructions. The EGFR antibody (ab2430) was used for RIP (Abcam, Cambridge, UK). The co-precipitated RNAs were detected by reverse transcription PCR and quantitative PCR. Total RNAs (input controls) and IgG were assayed simultaneously to demonstrate that the detected signals were the result of RNAs specifically binding to EGFR.

### Statistical methods

All experiments were independently repeated at least triplicate. Data were expressed as mean ± SEM. Differences between two independent groups were tested with the student *t* test. All statistical analyses were carried out using SPSS version 18.0 and presented with Graphpad prism software. Pearson correlation analysis was performed in calculating the correlation between Linc00152 and EGFR, The results were considered to be statistically significant at *P* < 0.05.

## Results

### Cytoplasm located Linc00152 was increased in gastric cancer

**Fig. 1:**
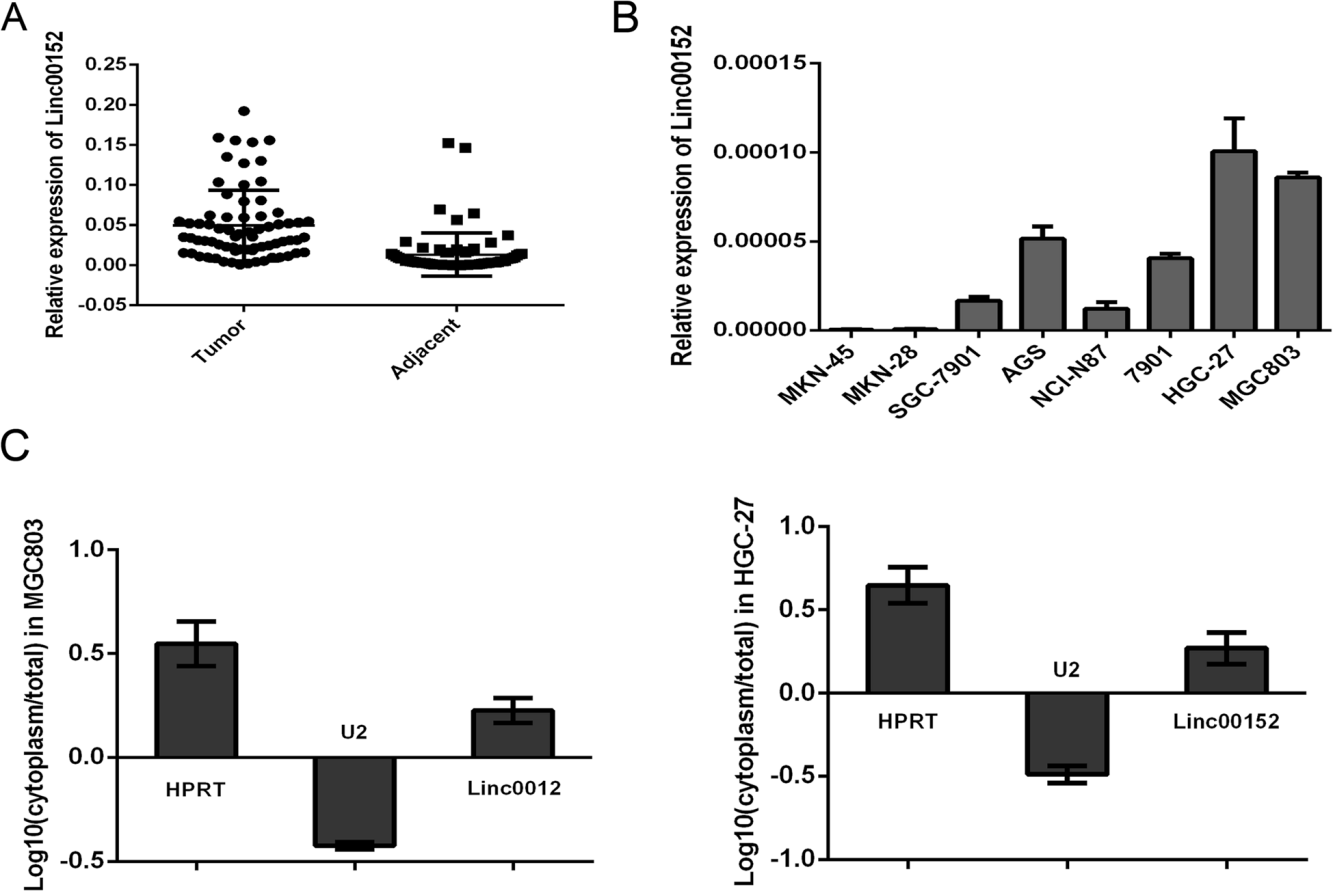
Cytoplasm located Linc00152 was increased in gastric cancer. **a** Relative expression level of Linc00152 in 72 pairs tissues obtained from gastric cancer patients. Data was presented with mean ± SEM. 18S was used as internal control. **b** Different expression of Linc00152 in gastric cancer cell lines. **c** The subcellular location and intensity of Linc00152 was examined by RT-PCR amplified with separated cytoplasm RNA and nuclear RNA in MGC803 and HGC-27 cells. HPRT was used as the control for cytoplasmic expression and U2 for nuclear. Data was presented with mean ± SEM

We first detected the expression of Linc00152 in 72 patients’ tissues suffering from gastric cancer. The qRT-PCR showed that the expression level of Linc00152 in gastric cancer tissues was significantly increased, compared with the corresponding adjacent non-normal tissues (Fig. 1a). In addition, we employed eight human gastric cancer cell lines to investigate the expression of Linc00152. We found that MGC803 and HGC-27 was expressed with a high abundance (Fig. 1b). We then detected the location of Linc00152 through a RT-PCR amplified with separated cytoplasm RNA and nuclear RNA in MGC803 and HGC-27 cells. We found that Linc00152 mainly located in the cytoplasm (Fig. 1c). According to the collected clinical information, the relevance between Linc00152 expression in gastric cancer tissues and clinicopathological characteristics is presented in Table 1. Here we found significant correlation with tumor size instead of tumor number, differentiation grade, TNM stage or metastasis.

**Table 1:** Clinical relevance of Linc00152 and EGFR and patients with gastric cancer

	Linc00152			EGFR		
Feather	Low	High	*P* value	Low	High	*P* value
All cases	36	36		36	36	
Age			0.326			0.637
< 60	11	15		16	18	
≥ 60	25	21		20	18	
Gender			0.422			0.808
Male	28	25		23	22	
Female	8	11		13	14	
Differentiation grade			0.616			0.864
Well	18	15		16	18	
Moderate	16	17		16	15	
Poorly	2	4		4	3	
Tumor size (cm)			**0.004**			**0.003**
≤ 5 cm	20	8		19	7	
> 5 cm	16	28		17	29	
Tumor Number			0.772			0.743
Solitary	28	29		30	31	
Multiple	8	7		6	5	
Metastasis			0.475			0.339
Yes	22	19		19	23	
No	14	17		17	13	
TNM stage			0.458			0.635
I-II	25	22		21	19	
III-IV	11	14		15	17	

*P* value in Bold indicated the significant analysis

### Linc00152 promoted cell proliferation in vitro

**Fig. 2:**
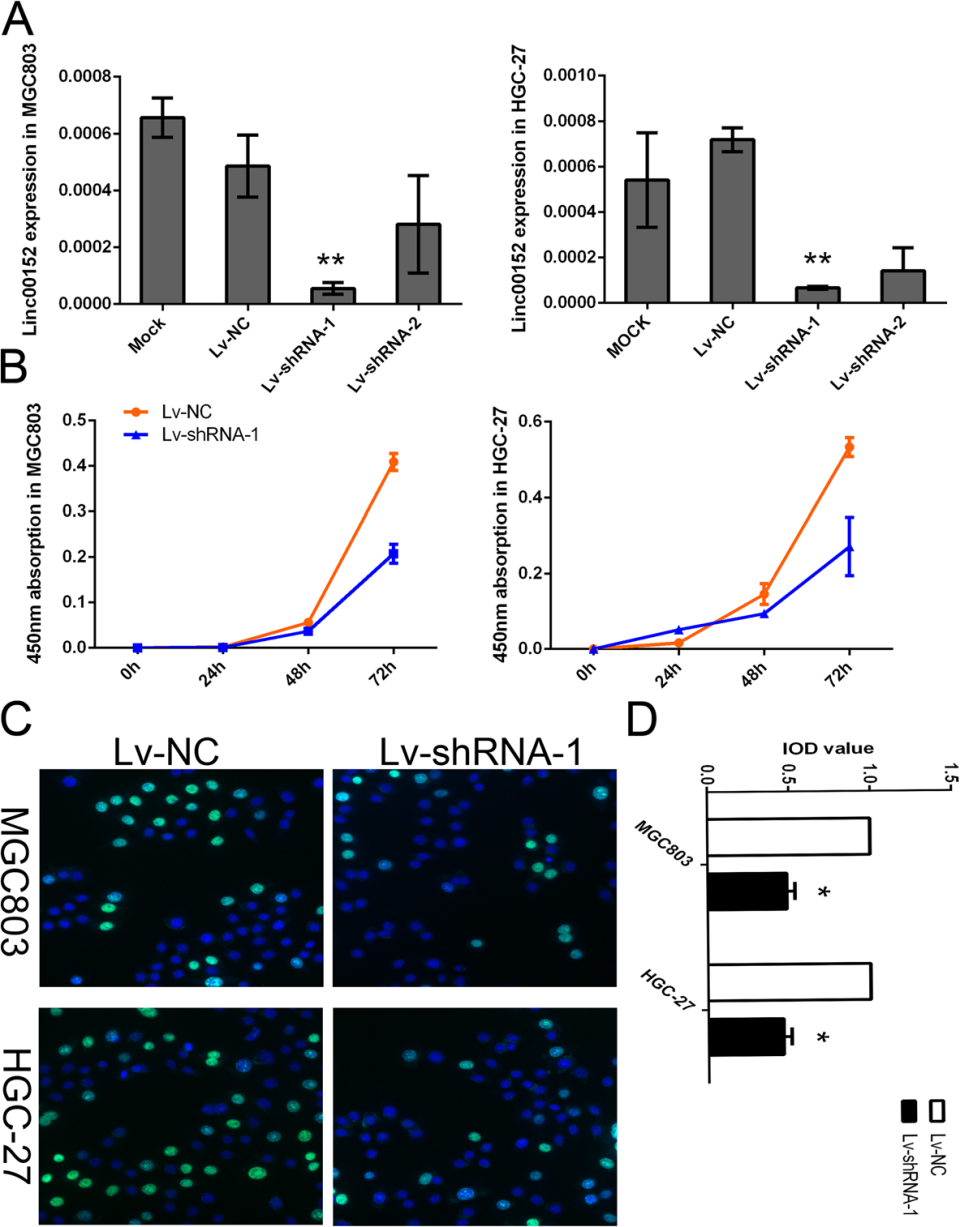
Decreased level of Linc00152 suppressed cell proliferation in vitro. **a** Expression level of Linc00152 in MGC803 and HGC-27 cells treated with shRNA. **b** The CCK8 assay detected in different time points including 0, 24, 48 and 72 h showed a decreased level of Linc00152 inhibited the growth of cell lines. Absorbance at 450 nm was presented as the mean ± SEM. **c** The EDU assay confirmed the functional role of Linc00152 in cell proliferation. **d ** The integral optical density value of cells treated with control plasmids was normalized to 100 %. All experiments were performed in triplicate and presented as the mean ± SEM. *indicates *P* < 0.05 while **indicated *P* < 0.01

The obvious correlation between Linc00152 expression and clinicopathological characteristics demonstrated that Linc00152 might play a vital role in the tumor growth of gastric cancer. We next selected MGC803 and HGC-27 to investigate the effects of shRNA-mediated knockdown of Linc00152 on cell proliferation. Two lncRNA-specific shRNAs were evaluated of their knockdown efficiency (Fig. 2a). We finally chose Lv-shRNA-1 in the following experiment due to a higher knockdown efficiency. CCK8 assay detected in different time point indicated that down-regulation of Linc00152 inhibited proliferation in both MGC803 and HGC-27 cells (Fig. 2b). Further EDU assay confirmed the results which also revealed that the decreased level of Linc00152 could cause a suppression of cell proliferation in both MGC803 and HGC-27 cells (Fig. 2c and d).

### Linc00152 induced a promotion of tumor growth in vivo

**Fig. 3:**
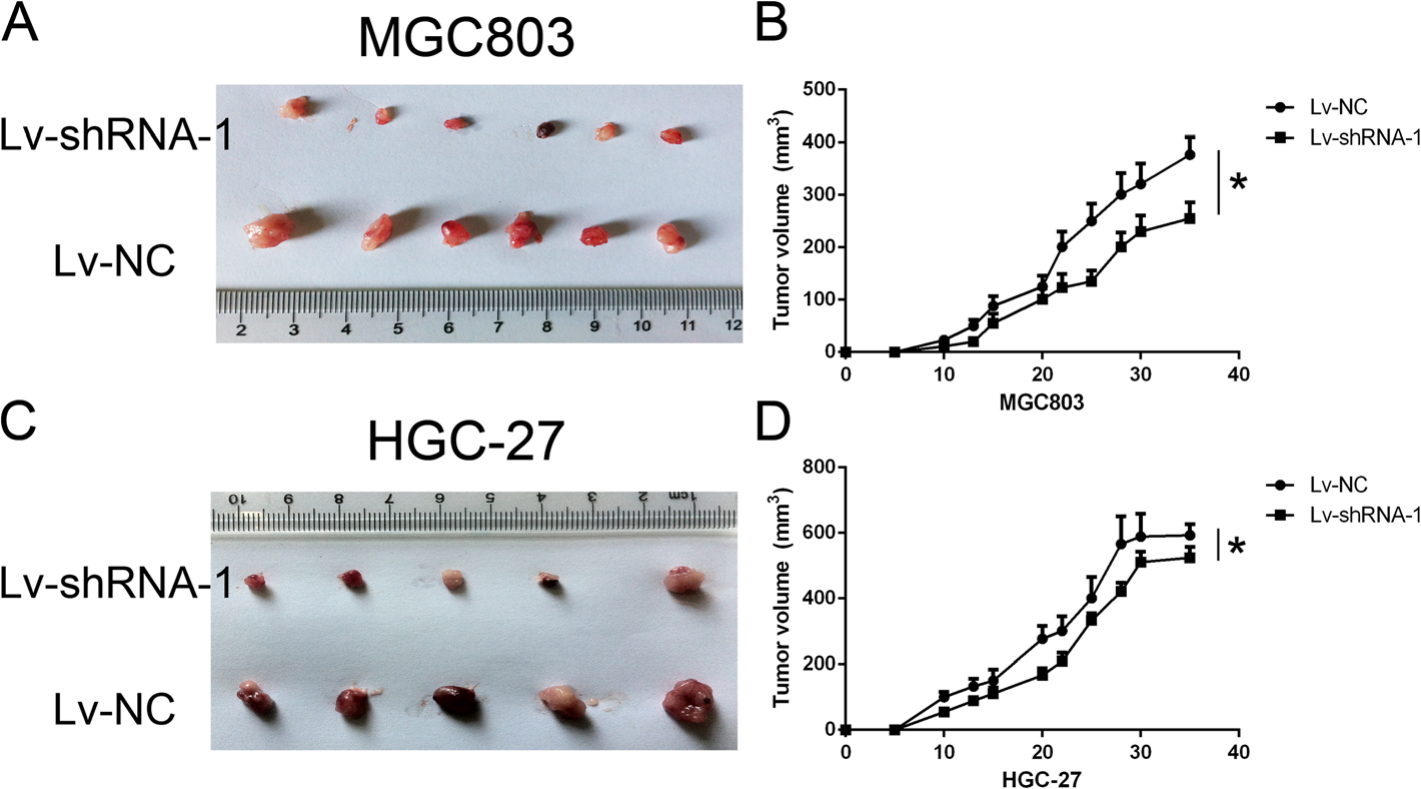
Down-regulation of Linc00152 suppressed tumor growth in vivo. **a**, **c** Tumor tissues obtained from Xenograft model in nude mice (at least 10 mice in each group). **a** panel presented MGC803 mice while (**c**) indicated HGC-27 cell lines. **b**, **d** Mice with established tumors were measured almost every three days and was presented in the right panel. **b** panel presented MGC803 mice while (**d**) indicated HGC-27 cell lines. *indicated *p* < 0.05

In order to confirm the effects of Linc00152 on tumorigenesis in vivo, we conducted a nude mice xenograft experiment by subcutaneously injected with MGC803 and HGC-27 cells stably knocked down for Linc00152 or a mock vector into flanks of 4–6 week-old BALB/C nude mice. As presented in Fig. 3a and c, we observed that tumor growth was significantly decreased compared with the mock. We also calculated the tumor volume nearly every three days, the growth curve presented in Fig. 3b and d demonstrated a remarkable suppression affection of cells treated with Linc00152 shRNA.

### Linc00152 constitutive activated PI3K/AKT signaling by direct binding with EGFR

**Fig. 4:**
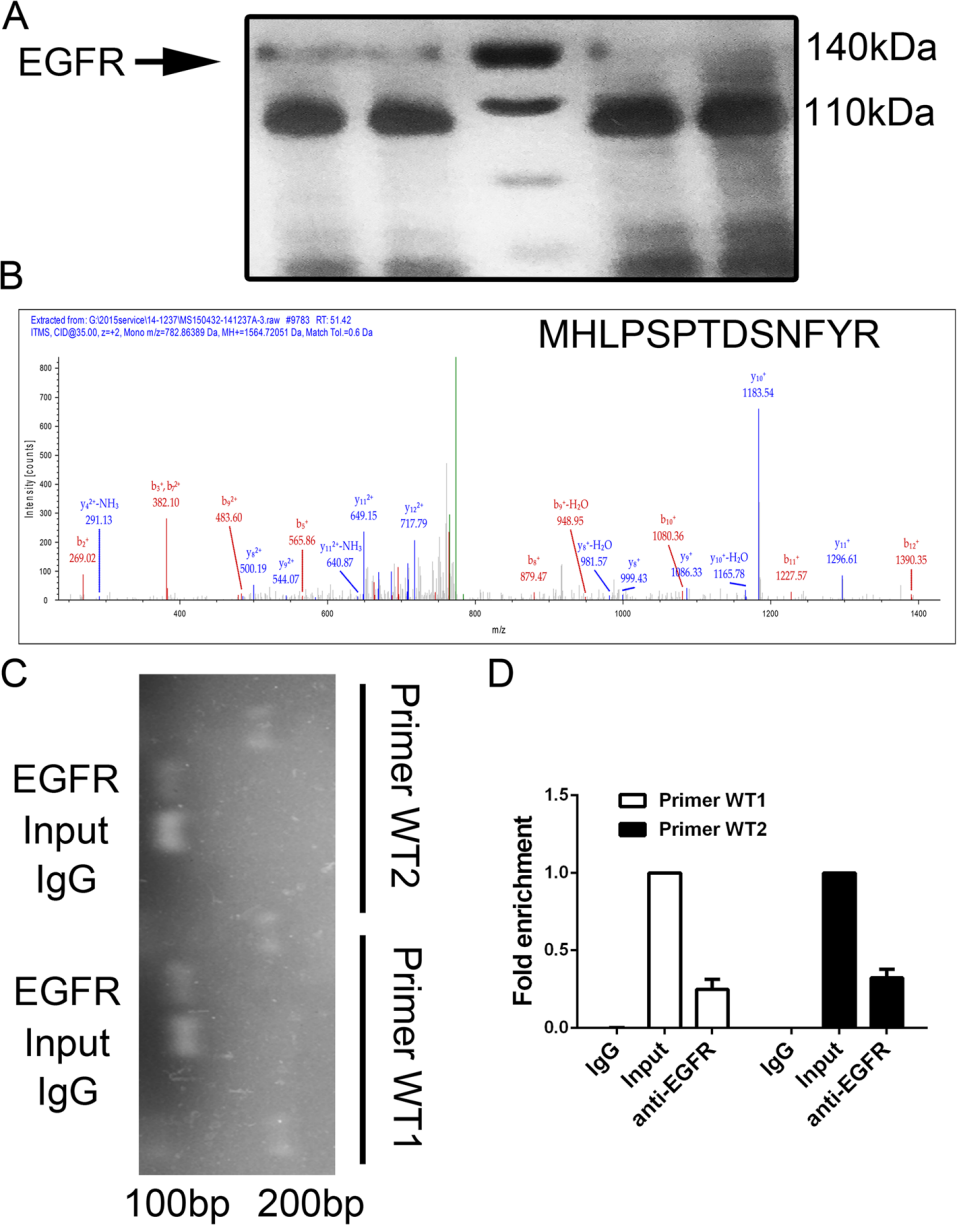
EGFR was identified as a binding target of Linc00152. **a** RNA pull-down experiment with MGC803 extract in different groups. The left two bands indicated Linc00152 wide type while the right two bands presented the antisense of Linc00152. **b** Mass spectrometry identification of target band of EGFR. The secondary ion mass spectrometry with the peptide sequence labeled was presented. **c**, **d** RIP assay was performed using EGFR antibody and was validated by agarose electrophoresis by using different primer. The band from the top to bottom presented DNA marker, RNA extracted with EGFR antibody treating, input (regard as a positive control) and IgG (negative control), respectively. Fold change enrichment was calculated comparing with the input in panel **d**. Data was presented with mean ± SEM

Linc00152 was reported to be up-regulated in multiple cancers and may play important roles in post-transcriptional regulation in cancer [17]. However, the specific signal pathway involved by the abnormal expression of Linc00152 still remained unknown. The RNA pull-down assay was applied to explore the potential binding protein. As presented in Fig. 4a, the protein located around 140 kDa was developed comparing with the antisense of Linc00152. Further mass spectrometry identification indicated that EGFR was the captured protein by Linc00152 (Fig. 4b). RNA immunoprecipitation (RIP) verified the specificity of this interaction, suggesting that Linc00152 could bind with EGFR and might regulate EGFR activity (Fig. 4c and d).

**Fig. 5:**
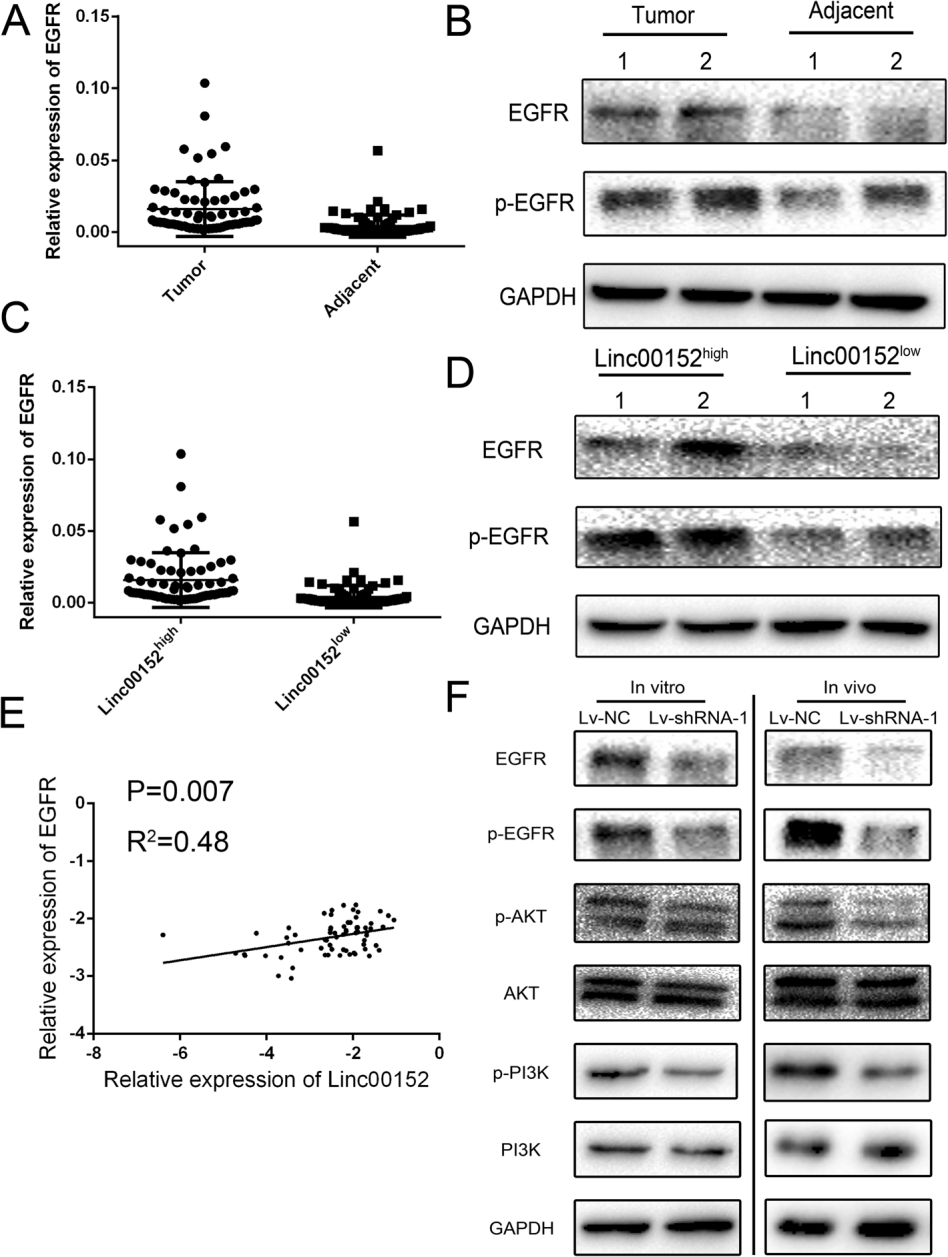
Linc00152 highly correlated with EGFR and constitutively activated PI3K/AKT signaling. **a** Different expression of EGFR mRNA in 72 pairs tissues from gastric cancer patients. **b** The protein expression level of EGFR in patients with gastric cancer. **c**, **d** Patients was divided into Linc00152^high^ and Linc00152^low^ groups based on the median of Linc00152 expression. The mRNA (panel **c**) and protein expression (panel **d**) of EGFR were compared in the two groups. **e** Pearson analysis was performed in calculating the correlation of Linc00152 and EGFR with log-transformed data. **f** The activation of PI3K/AKT signaling was measured by detecting the p-EGFR, p-PI3K and p-AKT in both cells line and tumors from Xenograft model. Data was presented with mean ± SEM

We next analyzed the mRNA and protein expression of EGFR in the tissues samples of patients mentioned above. We found a significant increased level of EGFR and p-EGFR in gastric cancers (Fig. 5a and b). We further divided the cancer tissues into Linc00152 high group and low group by using the median of Linc00152 expression as cutoff. Both the mRNA and protein investigation demonstrated that an elevated level of EGFR and p-EGFR accompany with Linc00152 (Fig. 5c and d). Pearson correlation analysis also presented a positive correlation between the expression of Linc00152 and EGFR (Fig. 5e). The expression of EGFR was also associated with tumor size as presented in Table 1. The traditional EGFR signaling pathway was detected by using samples obtained both in cells lines and xenograft tumor in mice. The inhibition of p-EGFR, p-AKT or p-PI3K was obtained in cells treated with Linc00152 shRNA which indicating that Linc00152 might cause a constitutive activation of EGFR signaling (Fig. 5f).

## Discussion

In our present study, we found that the average level of Linc00152 in GC tissues was significantly higher than those in corresponding non-tumor tissues. The high expression level of Linc00152 in GC patients was associated with tumor size. Similarly, it has been revealed that Linc00152 was up-regulated in liver caners and might be applied as a biomarker for the diagnosis. Our results confirmed the upregulation of Linc00152 in GC patients of our cohort. Based on the clinical characteristic analysis, we found that Linc00152 was highly associated with tumor size of GC patients instead of the metastasis or differentiation.

Although Linc00152 have been studied in a variety of physiological and pathological processes, such as liver cancer and pancreatic cancer [18], the possible role and associated molecular mechanism of Linc00152 in human gastric cancer remains to be clarified. Our results showed that Linc00152 knockdown could significantly inhibit gastric cancer cell proliferation both in vitro and in vivo. The RNA pull-down assay and RIP both confirmed that Linc00152 might be directly bind with EGFR which caused a constitutive activation of EGFR. LncRNA has been reported as an enhancer factor or suppression factor with specific protein through direct binding, for example, lncRNA-Dreh could combine with the intermediate filament protein vimentin and repress its expression [19].

EGFR belongs to the family of receptor tyrosine kinases (RTK) ErbB, which consisting of HER1/EGFR/ErbB1, HER2/Neu/ErbB2, HER3/ErbB3 and HER4/ErbB4 [20]. EGFR is overexpressed in various cancers, including non-small cell lung cancer, colorectal cancer, pancreatic cancer, esophagogastric cancer and gastric cancer as well [21]. EGFR is recognized as oncogenic driver in tumorigenesis and a target for cancer therapies. Researchers has identified that overexpression of EGFR could increase the proliferation of tumor cell through the PI3K-AKT signaling pathway and it is likely to be an independent predictor of poor prognosis [22]. So it is of great value to determine EGFR status to interpret future clinical trials properly using EGFR targeted agents.

## Conclusions

Our findings demonstrated the promotion effect of Linc00152 in vivo and vitro. Additionally, our results indicated that EGFR, contribute to the downstream regulation of Linc00152 in gastric cancer which may serve as potential targets for therapy in the future.

## Abbreviations

GAPDH: Glyceraldehyde 3-phosphate dehydrogenase
lncRNA: Long non-coding
RNAs qRT-PCR: Quantitative real time polymerase chain reaction
SDS-PAGE: sodium dodecyl sulphate polyacrylamide gel electrophoresis
10 % FBS: 10 % fetal bovine serum
EDU: 5-ethynyl-2′-deoxyuridine

## References

[1] Herszenyi L, Tulassay Z (2010). Epidemiology of gastrointestinal and liver tumors. Eur Rev Med Pharmacol Sci.

[2] Ferlay J, Soerjomataram I, Dikshit R, Eser S, Mathers C, Rebelo M (2015). Cancer incidence and mortality worldwide: sources, methods and major patterns in GLOBOCAN 2012. Int J Cancer.

[3] Catalano V, Labianca R, Beretta GD, Gatta G, de Braud F, Van Cutsem E (2009). Gastric cancer. Crit Rev Oncol Hematol.

[4] Vogiatzi P, Vindigni C, Roviello F, Renieri A, Giordano A (2007). Deciphering the underlying genetic and epigenetic events leading to gastric carcinogenesis. J Cell Physiol.

[5] Zhao W, Dong S, Duan B, Chen P, Shi L, Gao H (2015). HOTAIR is a predictive and prognostic biomarker for patients with advanced gastric adenocarcinoma receiving fluorouracil and platinum combination chemotherapy. Am J Transl Res.

[6] Pinheiro H, Bordeira-Carrico R, Seixas S, Carvalho J, Senz J, Oliveira P (2010). Allele-specific CDH1 downregulation and hereditary diffuse gastric cancer. Hum Mol Genet.

[7] Tang J, Zhuo H, Zhang X, Jiang R, Ji J, Deng L (2014). A novel biomarker Linc00974 interacting with KRT19 promotes proliferation and metastasis in hepatocellular carcinoma. Cell Death Dis.

[8] Tang J, Jiang R, Deng L, Zhang X, Wang K, Sun B (2015). Circulation long non-coding RNAs act as biomarkers for predicting tumorigenesis and metastasis in hepatocellular carcinoma. Oncotarget.

[9] Wang Y, Wu P, Lin R, Rong L, Xue Y, Fang Y (2015). LncRNA NALT interaction with NOTCH1 promoted cell proliferation in pediatric T cell acute lymphoblastic leukemia. Sci Rep.

[10] Zhuo H, Tang J, Lin Z, Jiang R, Zhang X, Ji J (2015). The aberrant expression of MEG3 regulated by UHRF1 predicts the prognosis of hepatocellular carcinoma. Mol Carcinog.

[11] Shen J, Siegel AB, Remotti H, Wang Q, Shen Y, Santella RM (2015). Exploration of Deregulated Long Non-Coding RNAs in Association with Hepatocarcinogenesis and Survival. Cancers (Basel).

[12] Merry CR, Forrest ME, Sabers JN, Beard L, Gao XH, Hatzoglou M (2015). DNMT1-associated long non-coding RNAs regulate global gene expression and DNA methylation in colon cancer. Hum Mol Genet.

[13] Yoon JH, Abdelmohsen K, Kim J, Yang X, Martindale JL, Tominaga-Yamanaka K (2013). Scaffold function of long non-coding RNA HOTAIR in protein ubiquitination. Nat Commun.

[14] Zhao J, Liu Y, Zhang W, Zhou Z, Wu J, Cui P, et al. Long non-coding RNA Linc00152 is involved in cell cycle arrest, apoptosis, epithelial to mesenchymal transition, cell migration and invasion in gastric cancer. Cell Cycle. 2015:1–12. doi:10.1080/15384101.2015.1078034. 10.1080/15384101.2015.1078034 PMC4825539 26237576

[15] Li Q, Shao Y, Zhang X, Zheng T, Miao M, Qin L (2015). Plasma long noncoding RNA protected by exosomes as a potential stable biomarker for gastric cancer. Tumour Biol.

[16] Tang W, Tang J, He J, Zhou Z, Qin Y, Qin J (2015). SLIT2/ROBO1-miR-218-1-RET/PLAG1: a new disease pathway involved in Hirschsprung's disease. J Cell Mol Med.

[17] Muller S, Raulefs S, Bruns P, Afonso-Grunz F, Plotner A, Thermann R (2015). Next-generation sequencing reveals novel differentially regulated mRNAs, lncRNAs, miRNAs, sdRNAs and a piRNA in pancreatic cancer. Mol Cancer.

[18] Li J, Wang X, Tang J, Jiang R, Zhang W, Ji J (2015). HULC and Linc00152 Act as Novel Biomarkers in Predicting Diagnosis of Hepatocellular Carcinoma. Cell Physiol Biochem.

[19] Huang JF, Guo YJ, Zhao CX, Yuan SX, Wang Y, Tang GN (2013). Hepatitis B virus X protein (HBx)-related long noncoding RNA (lncRNA) down-regulated expression by HBx (Dreh) inhibits hepatocellular carcinoma metastasis by targeting the intermediate filament protein vimentin. Hepatology.

[20] Patel R, Leung HY (2012). Targeting the EGFR-family for therapy: biological challenges and clinical perspective. Curr Pharm Des.

[21] Weng X, Zhang H, Ye J, Kan M, Liu F, Wang T (2015). Hypermethylated Epidermal growth factor receptor (EGFR) promoter is associated with gastric cancer. Sci Rep.

[22] Nam HJ, Ching KA, Kan J, Kim HP, Han SW, Im SA (2012). Evaluation of the antitumor effects and mechanisms of PF00299804, a pan-HER inhibitor, alone or in combination with chemotherapy or targeted agents in gastric cancer. Mol Cancer Ther.

