# Erratum to: Linc00152 promotes proliferation in gastric cancer through the EGFR-dependent pathway

**DOI:** 10.1186/s13046-015-0262-2

**Published:** 2016-02-09

**Authors:** Jianping Zhou, Xiaofei Zhi, Linjun Wang, Weizhi Wang, Zheng Li, Jie Tang, Jiwei Wang, Qun Zhang, Zekuan Xu

**Affiliations:** Department of General Surgery, The First Affiliated Hospital of Nanjing Medical University, Nanjing, Jiangsu P.R. China; Department of Gastrointestinal Surgery, The Affiliated Yixing Hospital of Jiangsu University, Yixing, Jiangsu P.R. China; Department of General Surgery, The Affiliated Hospital of Nantong University, Nantong, Jiangsu P.R. China

Unfortunately, the original version of this article [1] contained several errors:

- Table 1 included incorrect EGFR data. The EGFR data shown were obtained from patients with hepatocellular carcinoma rather than gastric cancer as indicated. These data were erroneously included during the drafting of the manuscript. The correct version of Table 1 showing the EGFR data obtained from gastric cancer patients can be found below. In addition, the correct version of Table 1 below does not show the TNM stage data reported in the original Table; as a result, the last sentence in the section “Cytoplasm located Linc00152 was increased in gastric cancer” should read “Here we found significant correlation with tumour size instead of tumour number, differentiation grade or metastasis.”

**Table 1:** Clinical relevance of Linc00152 and EGFR and patients with gastric cancer

	Linc00152			EGFR		
Feather	Low	High	*P* value	Low	High	*P* value
All cases	36	36		36	36	
Age			0.326			0.637
<60	11	15		13	13	
≥60	25	21		23	23	
Gender			0.422			0.061
Male	28	25		23	30	
Female	8	11		13	6	
Differentiation grade			0.616			0.616
Well	18	15		15	18	
Moderate	16	17		17	16	
Poorly	2	4		4	2	
Tumor Size(cm)			0.004			0.016
≤5 cm	20	8		19	9	
>5 cm	16	28		17	27	
Tumor Number			0.772			0.384
Solitary	28	29		30	27	
Multiple	8	7		6	9	
Metastasis			0.475			0.812
Yes	22	19		20	21	
No	14	17		16	15
- In Figure 5, panel a was replicated as panel c. The correct Figure 5 can be found below.

**Fig. 5:**
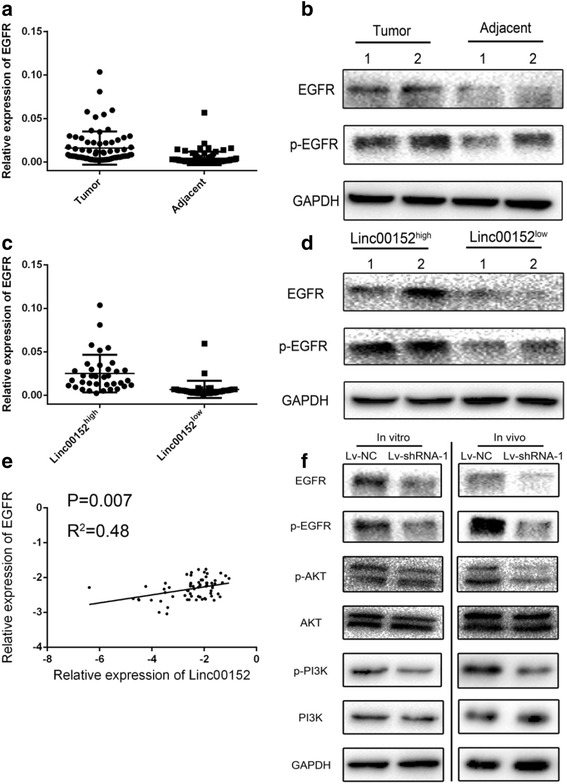
Linc00152 highly correlated with EGFR and constitutively activated PI3K/AKT signaling. **a** Different expression of EGFR mRNA in 72 pairs tissues from gastric cancer patients. **b** The protein expression level of EGFR in patients with gastric cancer. **c**, **d** Patients was divided into Linc00152^high^ and Linc00152^low^ groups based on the median of Linc00152 expression. The mRNA (panel **c**) and protein expression (panel **d**) of EGFR were compared in the two groups. **e** Pearson analysis was performed in calculating the correlation of Linc00152 and EGFR with log-transformed data. **f** The activation of PI3K/AKT signaling was measured by detecting the p-EGFR, p-PI3K and p-AKT in both cells line and tumors from Xenograft model. Data was presented with mean ± SEM
- The acknowledgements were given as “This work was supported by the Foundation for the Talents in Six Kinds of Profession of Jiangsu Province (JSGF2015D2914 to J.Z.); Natural Science Foundation of Jiangsu Province (BK20151136 to J.Z.).” Instead of “This work was supported by the Foundation for the Talents in Six Kinds of Profession of Jiangsu Province (WSW-075 to J.Z.); Natural Science Foundation of Jiangsu Province (BK20151136 to J.Z.); National Natural Science Foundation of China (81272712, 81072031 to Z.X.); the National Natural Science Foundation Project of International Cooperation (NSFC-NIH, 812111519 to Z.X.); the Program for Development of Innovative Research Team in the First Affiliated Hospital of NJMU, the Priority Academic Program Development of Jiangsu Higher Education Institutions (PAPD, JX 10231801 to Z.X.)”.

The correct Table 1 and Fig. 5c, as well as the acknowledgements, can be found below correctly.
